# The Experimental Study on Geometric Calibration and Material Discrimination for In Vivo Dual-Energy CT Imaging

**DOI:** 10.1155/2019/7614589

**Published:** 2019-05-22

**Authors:** Gang Deng, Mianyi Chen, Peng He, Xing Wang, Xiaochuan Wu, Xiaodong Guo, Pengcheng Li, Biao Wei, Kang An, Xiaolin Zheng, Peng Feng

**Affiliations:** ^1^The Key Laboratory of Rheological Science and Technology of the Education Ministry of China, Chongqing University, Chongqing 400044, China; ^2^The Key Lab of Optoelectronic Technology and Systems of the Education Ministry of China, Chongqing University, Chongqing 400044, China; ^3^ICT Engineering Research Center of Ministry of Education, Chongqing University, Chongqing 400044, China; ^4^Collaborative Innovation Center for Brain Science, Chongqing University, Chongqing 400044, China

## Abstract

Photon-counting detector (PCD) can identify absorption features in the multiple ranges of photon energies, which has a great potential in material discrimination. In this paper, we focused on in vivo dual-energy CT imaging to characterize different biomedical compositions. The precision of material decomposition in post-reconstruction space depends on the quality of reconstructed CT images; we used the locally linear embedding (LLE) based online geometric calibration method and GPU-based reconstruction toolbox to reconstruct high-quality CT images. Then, we performed the real experiment and studied materials decomposition with basis material model to discriminate soft tissue and cortical bone of small animal. Finally, the experimental results demonstrated that the proposed method could reconstruct small animal CT images with more slim structures and details, and improve the precision of materials decomposition in dual-energy CT imaging.

## 1. Introduction

X-ray computed tomography (CT) system has been widely applied in clinical and preclinical applications [1, 2]. However, the conventional CT system does not have the capability to resolve the energy of every single photon interacting with the sensor layer. With the development of x-ray detection technique, the photon-counting detector (PCD) could use selectable energy thresholds (i.e., selective energy window/range) to identify an absorption feature in the available x-ray energy ranges [3, 4]. Thus, it is more efficient to employ PCD in dual-energy CT imaging, which has a great potential in biomedical material discrimination [5–9].

Current dual-energy CT technique has been used to decompose materials in medicine. Alvarez and Macovski developed the attenuation coefficient function based on dual-energy measurements to separate the photoelectric effect and Compton scattering in pre-reconstruction space [10]. Heismann et al. used density and atomic number as functions of attenuation values to separate the photoelectric effect and Compton scattering in post-reconstruction space [11]. Kalender et al. proposed dual-energy material decomposition method based on physical property of basis material in pre-reconstruction space [12]. Liu X. et al. introduced basis material decomposition method in post-reconstruction space [13].

The precision of material decomposition in post-reconstruction space highly depends on the quality of reconstructed CT images. Therefore, the reconstruction method and geometric calibration are the key elements. In this paper, we use iterative method to reconstruct dual-energy CT image, which can get high quality CT images with less projections or noisy projections [14–18]. In order to accelerate the iterative reconstruction, we used the GPU-based ASTRA (All Scale Tomographic Reconstruction Antwerp) toolbox [19–21] that could be used in Matlab and Python. Moreover, the ASTRA toolbox could calculate the projection and reconstruction with any system geometries by building the coordinates of x-ray source and detector with vectors.

The accurate geometric parameters are the key factors for the CT image reconstruction. The geometric calibration methods could be divided into phantom based calibration methods and online calibration methods. The phantom-based calibration methods calculate the geometric parameters using the projection of the calibration phantom and CT imaging geometry, while the online calibration methods could calibrate imaging geometry without any scanning objects. Thus, we use the Locally Linear Embedding (LLE) [22] based geometric calibration method [23–25] in this study. The LLE-based geometric calibration method is an online geometric calibration method which is suitable for any CT systems. First, we calculate the re-projected projection with reconstructed images by sampling the geometric parameters in the sampling range. Then, we find the two nearest re-projected projections and calculate the weight coefficients with LLE. Finally, we update the geometric parameters and reconstruct the CT images.

In this paper, we focused on in vivo dual-energy CT imaging for biomedical material discrimination. To improve the precision of materials decomposition by the basis material decomposition method in post-reconstruction space, we employed the LLE-based geometric calibration method and GPU-based reconstruction toolbox to reconstruct high-quality CT images. In the second section, we introduce the characteristics of research materials and methods. In the third section, we present our experimental results on geometric calibration and material discrimination. In the last section, we discuss the related issues and conclude the paper.

## 2. Method and Material

### 2.1. Sample Preparation

**Figure 1:**
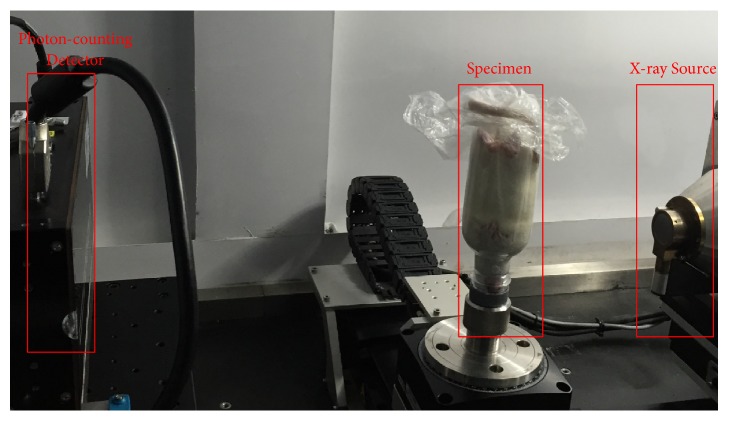
The micro-CT system based on x-ray PCD.

The experimental protocol is carried out with approval from the Chinese Army Medical University Animal Care and Use Committee. The adult Sprague Dawley rats (350~400g) were purchased from Animal Experiment Center of Medical University of Chongqing. The rat was fed in cleanliness level independent ventilation cage (IVC0200, Hongteng Technology, Shenzhen, China) in a constant temperature laboratory (23°C). The rats were anesthetized with urethane (1.2g/kg. 20%. ip). Five to ten minutes later, we inverted the rat into an inverted plastic bottle, as shown in Figure 1. The rat head lied on the bottom of the bottle, and the rat's butt was facing up. The bottle was placed on the scanning rotation table. All the design ensured that the heart of the rat was located at the precise level for successful imaging. All experiments were strictly carried out in accordance with the Experimental Animal Management Regulations of China National Science and Technology Council.

### 2.2. Dual-Energy CT Imaging System

We constructed a micro-CT system, which employed a broad spectral x-ray source and a PCD, as shown in Figure 1. The x-ray tube (L10101, Hamamatsu Photonics, Japan) has a focal spot of 5*μ*m, and the voltage ranges from 20kVp to 100kVp. The detector manufactured by X-Counter Corporate defines the finest spatial resolution and fastest imaging speed, and each individual detector cell has two energy thresholds to select and record incoming photons. The PCD contains 2048×64 pixels with 100*μ*m pixel size, and the detection range of x-ray energy is from 15keV to 250keV. In the experimental study, we scanned an adult Sprague Dawley rat specimen. The energy thresholds of PCD were set to 15keV and 60keV; the tube voltage and current are 100kVp and 70*μ*A, respectively. The angular scanning range is 360° with 0.72° increment, producing 500 projections in low- and high-energy spectrum, respectively. The source to detector (SDD) is 480mm, and the source to objection distance (SOD) is 160mm.

### 2.3. Geometry Calibration and Reconstruction Method

#### 2.3.1. Reconstruction Method

The foundation mathematical model for iterative reconstruction can be expressed as the following system of linear equations:

(1) $$\begin{array}{c} Au=b, \end{array}$$

where **u** = (*u*_1_, *u*_2_,…,*u*_*J*_)^*T*^ is an image as a *J* dimensional vector, **b** = (*b*_1_, *b*_2_,…,*b*_*M*_)^*T*^ is projection data, and **A** = (*a*_*jm*_) is a system matrix determined by the geometric parameters. In this mathematical model, the projection matrix and the projection data are known, and the CT reconstruction could be considered to calculate all the pixel values of CT image by solving the linear equations.

CT reconstruction is a time consuming procedure, especially for iterative reconstruction method. In order to accelerate CT reconstruction speed, we used the ASTRA-toolbox which a GPU-based CT reconstruction toolbox. The iterative reconstruction method could reconstruct high-quality CT images with less noise than analytic reconstruction method. Therefore, we are using the iterative reconstruction method. The ASTRA-toolbox only provides CGLS (Conjugate Gradient Least Squares) and SIRT (Simultaneous Iterative Reconstruction Technique) for 3D iterative reconstruction, and CGLS could reconstruct better images within less iteration. Finally, we perform CGLS reconstruction; the description of CGLS is in the following.

First, initialize the variables for the CGLS, which are

(2) u0≔0;d0=b;r0=ATb;p0=r0;t0=Ap0,

where *u*_0_ is the initialized reconstructed image, *b* is the projection data, *A* is the projection matrix, *d*_0_, *r*_0_, *p*_0_, *t*_0_are the initialized intermediate variables. Then, for k=1,2,… until stopping criterion is satisfied:

(3) ∂k=rk−12tk−12uk=uk−1+∂kpk−1dk=dk−1−∂ktk−1rk=ATdkβk=rk2rk−12pk=rk+βkpk−1tk=Apk,

where ∂_*k*_, *β*_*k*_ are the iteration parameters, *u*_*k*_ and *u*_*k*−1_ are the reconstructed image in the current iteration and last iteration, *d*_*k*_, *r*_*k*_, *p*_*k*_, *t*_*k*_ are the intermediate variables.

#### 2.3.2. The LLE-Based Geometric Calibration Method

The LLE-based geometric calibration method has been detailed described in the previous papers [23–25]; here we present the implementation of the LLE-based geometric calibration method as the following steps:

**Step 1 .**

Initialize a parameter vector **P** and perform the image reconstruction; sample the parameter vector densely in the parametric ranges for each projection as

(4) $$\begin{array}{c} {\tilde{P}}_{m}=\left(\;p_{m1},p_{m2},\cdots ,p_{mn}\;\right). \end{array}$$

**Step 2 .**

Calculate the re-projected projections ${\tilde{b}}_{m}$ with the sampled parameter vector and the reconstructed images.

**Step 3 .**

Find the two nearest neighbors of the original projection vector **b** in the re-projected projections ${\tilde{b}}_{m}$ according to the projection errors

(5) $$\begin{array}{c} d_{m}={\left\|\;b-{\tilde{b}}_{m}\;\right\|}_{2}^{2}. \end{array}$$

Then, the original projection vector **b** is linearly represented with the nearest neighbors ${\tilde{b}}_{k}$ as

(6) $$\begin{array}{c} b=\overset{K}{\underset{k=1}{\sum }}w_{k}{\tilde{b}}_{k}, \end{array}$$

where *w*_*k*_ is the linear representation weights.

**Step 4 .**

Calculate the weight coefficients by solving the following linear equations:

(7) $$\begin{array}{c} \underset{k}{\sum }c_{mk}w_{k}=1, \end{array}$$

where *c*_*mk*_ is the local covariance matrix.

**Step 5 .**

Update the parameter vector with the weight coefficients and the sampled vector ${\tilde{P}}_{k}$ of the two nearest re-projections ${\tilde{b}}_{k}$ as

(8) $$\begin{array}{c} P=\overset{K}{\underset{k=1}{\sum }}w_{k}{\tilde{P}}_{k}. \end{array}$$

**Step 6 .**

Reconstruct the CT images and evaluate the quality of the CT images. If image quality meets some criterions, output the CT images and updated parameters. Otherwise, return to Step 2 for next calibration iteration.

For PCD based micro-CT system, the scanning object is fixed on the stable rotation stage, which means the geometric parameters will not change during the scanning. Therefore, we only need to calibrate the rotation stage center offset in horizontal direction, rotation stage center offset in vertical direction and detector in-plane rotation by the LLE-based online geometric calibration method and the projection of the scanning object.

### 2.4. Material Discrimination Method

PCD with selectable thresholds could adjust each pixel to record different energy photons. A given selectable threshold *T* corresponding to incident photon energy can be *E*(*T*), and the energy distribution function of an x-ray source is expressed as *S*(*E*); we have the photon number received by the PCD with an energy range (*E*(*T*_1_), *E*(*T*_2_))

(9) $$\begin{array}{c} I\left(\;E\;\right)=\overset{E\left(T_{2}\right)}{\underset{E\left(T_{1}\right)}{\int }}S\left(\;E\;\right)dE, \end{array}$$

where the two selectable thresholds *T*_1_ and *T*_2_ corresponding to photon energies meet *E*(*T*_1_) < *E*(*T*_2_). After the x-ray source interacted with an object, the projection Pr*o*(*E*) is the integral of the linear attenuation coefficient distribution along an x-ray path, which can be measured by intensities *I* and *I*_0_

(10) ProE−ln⁡IEI0E=ln⁡∫ET1ET2SEdE−ln⁡∫ET1ET2SEexp∫LμE,ldldE.

Therefore, we can set different selectable threshold *T* to measure projection data with different energy ranges. In this paper, we take two distinct x-ray energy spectra measurements by the micro-CT system based on PCD; the high energy and low energy projections can be expressed as

(11) ProEL=−ln⁡IELI0ELProEH=−ln⁡IEHI0EH,

where high energy spectrum corresponding to projection is Pr*o*(*E*_*H*_), and low energy spectrum corresponding to projection is Pr*o*(*E*_*L*_). Then we can reconstruct two CT images using projection datasets with two energy ranges.

In order to discriminate soft tissue and bone material of vivo small animal from two distinct x-ray energy spectra measurements, here we used the basis material decomposition method in post-construction space. The integrated attenuation coefficient can be expressed as the product of two material components:

(12) $$\begin{array}{c} \mu \left(\;E\;\right)=a_{1}\mu_{1}\left(\;E\;\right)+a_{2}\mu_{2}\left(\;E\;\right), \end{array}$$

where *a*_1_ and *a*_2_ are decomposition coefficients of two materials, *μ*_1_(*E*) and *μ*_2_(*E*) are the linear attenuation coefficients of two materials.

To effectively decompose the two biomedical compositions, basis materials should be selected close to the atomic number of bone and soft tissue. The main component of bone is calcium carbonate, and the muscular tissues are mainly composed of water and protein; therefore we selected water component and calcium component as two basis materials to analyze soft tissue and bone material of small animal, and the linear attenuation coefficients in the high energy and low energy range can be expressed as

(13) μEL=a1μ1EL+a2μ2ELμEH=a1μ1EH+a2μ2EH,

and equation (13) can be written in matrix form as

(14) $$\begin{array}{c} \left(\begin{array}{cc} \mu_{1}\left(E_{L}\right) & \mu_{2}\left(E_{L}\right)\\ \mu_{1}\left(E_{H}\right) & \mu_{2}\left(E_{H}\right) \end{array}\right)\left(\begin{array}{c} a_{1}\\ a_{2} \end{array}\right)=\left(\begin{array}{c} \mu \left(E_{L}\right)\\ \mu \left(E_{H}\right) \end{array}\right), \end{array}$$

where $U=\left(\begin{array}{cc} \mu_{1}(E_{L}) & \mu_{2}(E_{L})\\ \mu_{1}(E_{H}) & \mu_{2}(E_{H}) \end{array}\right)$ is the material composition matrix, which represents the linear attenuation coefficients of two basis materials in high and low energy ranges. Since the attenuations of water and calcium are irrelevant, *A* is a reversible matrix. Then, we can calculate the decomposition coefficients of two materials in matrix form as follows:

(15) a1a21μ1ELμ2EH−μ1EHμ2EL·μ2EH−μ2EL−μ1EHμ1ELμELμEH.

Finally, we can obtain the linear attenuation characteristics of two materials and distinguish soft tissue and bone materials.

## 3. Results

### 3.1. Geometric Calibration and Reconstruction

In this section, we will calibrate the geometric parameters using LLE-based geometric calibration method, and the calibration parameters are rotation stage center offset in horizontal direction, rotation stage center offset in vertical direction, and detector in-plane rotation. With the powerful calculation ability of Graphic card, the geometric calibration procedure could be extremely accelerated. In order to demonstrate the acceleration ability, we processed the correction procedure with the algorithms based on GPU including projection generation and image reconstruction. The total cost time of geometric calibration protocol based on CPU is 360 minutes while the computation cost based on GPU is only 1.27 minutes; the acceleration rate is 283 times.

**Figure 2:**
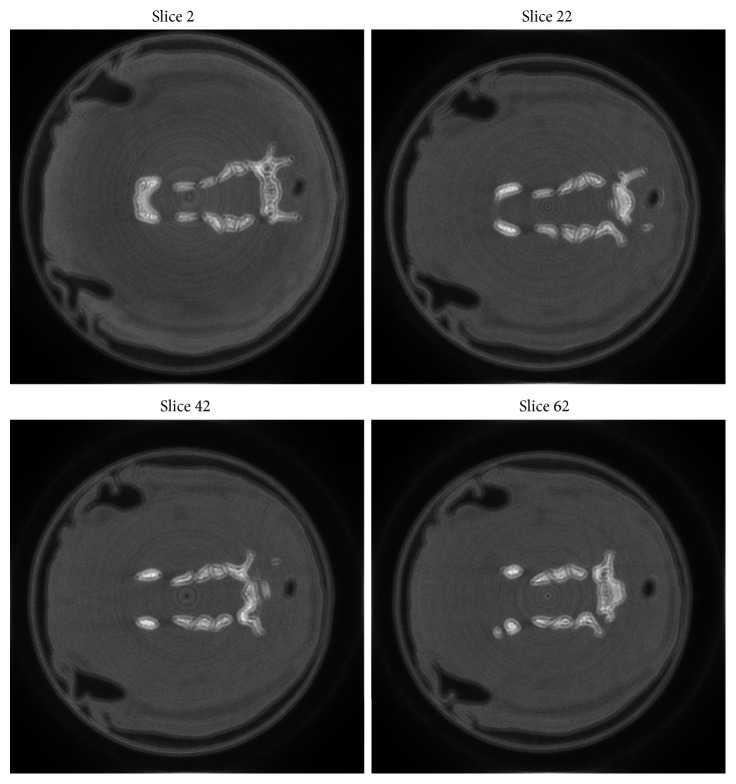
The reconstructed images before geometric calibration, display window: [0 0.5].

**Figure 3:**
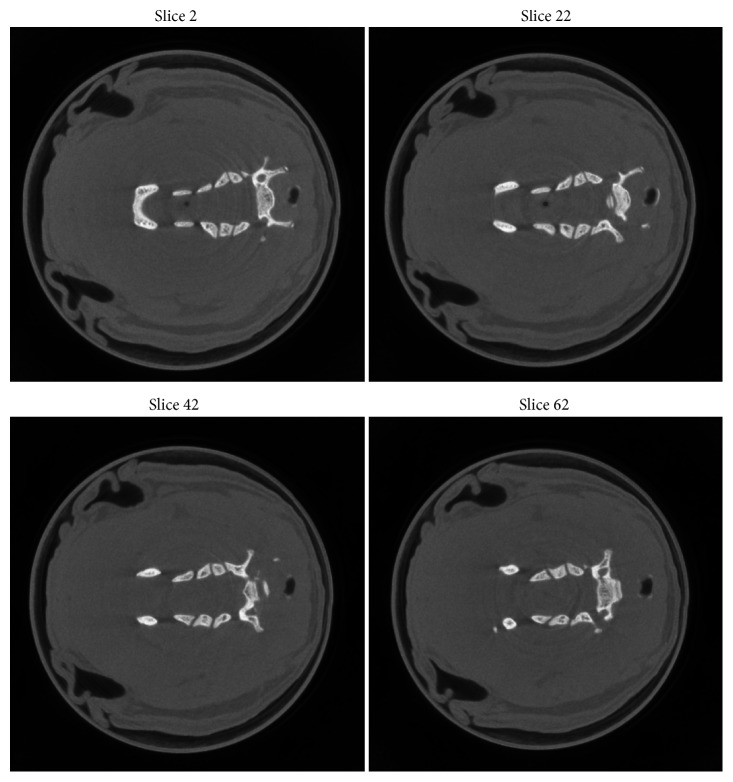
The reconstructed images after geometric calibration, display window: [0 0.5].

The original geometric parameters are shown in Table 1, and the reconstructed images before geometric calibration are shown in Figure 2; it is clearly seen that there are some artifacts in the reconstructed images. The reconstructed images after geometric calibration are shown in Figure 3, the proper parameters' sampling range and sampling rate are summarized in Table 2, and the calibrated parameters are also shown in Table 1. From Figure 3, the artifacts of the reconstructed images after geometric calibration have been removed.

According to the study [26], we used the Total Variation of the reconstructed images at different calibration iterations to quantify calibration results. The smaller the Total Variation is, the less geometric the artifacts are. The Total Variation of slice 22 at different calibration iterations is shown in Table 3.

### 3.2. Biomedical Material Discrimination

**Figure 4:**
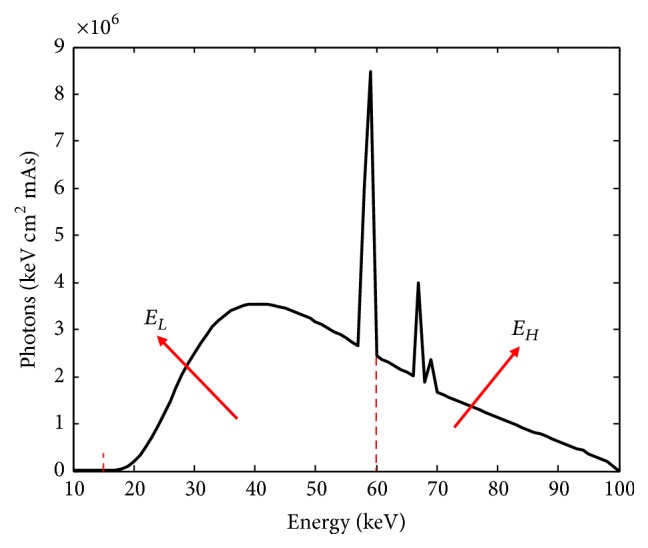
Source photon emission spectra.

**Figure 5:**
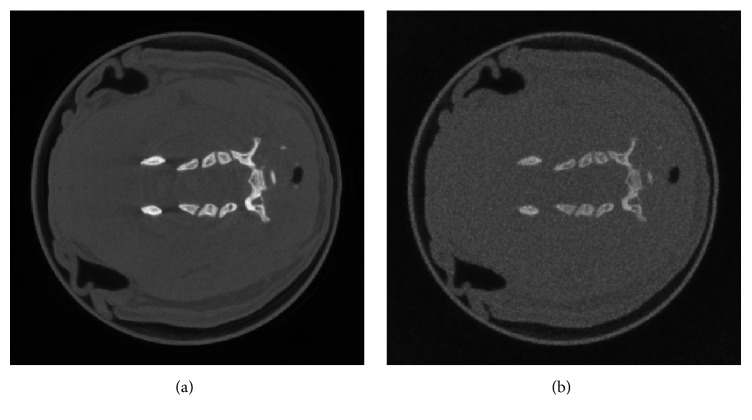
Reconstructed CT images at one slice. (a) Image at energy of 15-60keV, (b) Image at energy of 60-100keV. Display window: [0 0.5].

For material decomposition experiment, we analyzed the chest of the rat with the dual-energy CT imaging system and obtained two sets of projection data in low- and high-energy spectrum, respectively. According to the specific x-ray source in our experiment, we used a free-of-charge software program (SpekCalc) [27] to calculate x-ray spectra from tungsten anode tubes, and the simulated spectrum is shown in Figure 4. The energy range is from 15keV to 100keV, and we marked the two energy ranges in Figure 4. Then we used GPU based ASTRA reconstruction toolbox to reconstruct the rat specimen CT images; one slice images with two energy ranges are shown in Figure 5.

**Figure 6:**
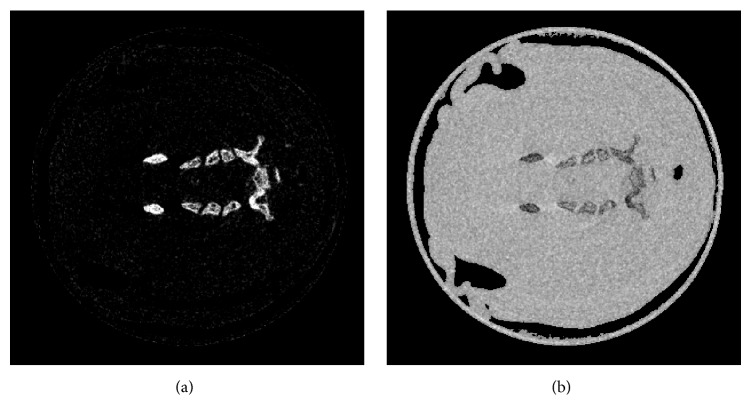
Reconstructed images after material decomposition. (a) The reconstructed calcium component image, display window [0 0.2]; (b) The reconstructed water component image, display window [0 2].

Then two material decomposition coefficient linear integrals can be calculated using Eq. (15). Finally, we could decompose the calcium and water materials images, which are shown in Figure 6. From Figure 6, we can discriminate two material compositions with own distinct features. The water component image highlights the soft tissue of the mouse, and calcium component image demonstrates the cortical bone of the mouse.

## 4. Conclusion and Discussion

There are some issues worthy of further discussion. In geometry calibration, a closely-related issue is the parametric sampling rate for re-projection. The greater the sampling rate is, the more accurate the calibration results will be, but the higher computational cost will be bigger. Therefore, we need to choose a proper sampling rate to balance between calibration results and computational cost. Although the smaller sampling rate could calibrate the geometric parameters more precisely, the sampling rate could not be set to infinite small, which depends on the memory size of graphic card. According to our experimental analysis, the number of selected sampling points is 20.

For material discrimination, we used the basis material method in post-reconstruction space to decompose two different materials from two distinct x-ray energy spectra measurements. The advantages in PCD technology allow for CT systems to identify absorption features in the multiple ranges of photon energies; therefore we will measure more energy spectrum data to discriminate multi-material in a follow-up research. With the development of novel contrast agents, we can also take contrast agent imaging to improve contrast resolution of CT image and characterize different soft tissues.

In conclusion, this study focuses on in vivo dual-energy CT imaging for biomedical material discrimination. We used the LLE-based geometric calibration method and GPU-based reconstruction toolbox to obtain high-quality reconstructed CT images and chose the basis material decomposition method in post-construction space to decompose soft tissue and bone compositions of rat specimen. Finally, the experimental results demonstrated the advantages of the PCD for material discrimination, which could establish guidelines for in vivo dual-energy CT imaging.

## Figures and Tables

**Table 1:** Original geometric parameters and calibrated geometric parameters.

Parameters	Original	Calibrated
Horizontal rotation center offset	0 mm	0.75 mm

Vertical rotation center offset	0 mm	0.51mm

Detector in-plane rotation	0°	0.12°

**Table 2:** Geometric parameter error ranges and sampling rates.

Parameters	Sampling range	Sampling rate
Horizontal rotation center offset	[-1mm 1mm]	0.01 mm

Vertical rotation center offset	[-1mm 1mm]	0.01mm

Detector in-plane rotation	[-0.5° 0.5°]	0.01°

**Table 3:** The Total Variation of slice 22 at different calibration iterations.

Iteration	0	1	2
Total Variation	0.0031	0.0020	0.0018

## Data Availability

The data used to support the findings of this study are available from the corresponding author upon request.
